# Kinetics of Circulating Damage-Associated Molecular Patterns in Sepsis

**DOI:** 10.1155/2015/424575

**Published:** 2015-06-16

**Authors:** Takahiro Miki, Toshiaki Iba

**Affiliations:** Department of Emergency and Disaster Medicine, Juntendo University Graduate School of Medicine, 2-1-1 Hongo, Bunkyo-ku, Tokyo, Japan

## Abstract

Circulating levels of conventional biomarkers and damage-associated molecular patterns were examined in 30 severe sepsis patients (20 survivors and 10 nonsurvivors). Plasma levels of interleukin 6, CRP, and procalcitonin reached their peaks on Day 0 (onset of sepsis) or Day 1 and declined rapidly thereafter despite the persistent severity. In contrast, elevated levels of histone H3, nucleosome, and high-mobility group protein Box 1 remained for longer periods of time. The peak level of histone H3 in the nonsurvivors was higher than that of the survivors (*p* < 0.05 on Day 7). The cutoff value of the histone H3 on Day 7 for death was 0.08 AU and the area under the receiver operating characteristic curve showed discriminative powers of 0.74. Measurement of circulating levels of the histone H3 provides additional information to that of the conventional indicators of inflammation for determining the severity of sepsis.

## 1. Introduction

It is important to identify reliable biomarkers to evaluate the severity of sepsis. More than a hundred markers have been examined so far; however, only a limited number have remained as candidates [1, 2]. Interleukin 6 (IL-6), a representative proinflammatory cytokine, has been reported as one of the most useful markers to determine the severity of sepsis. Plasma levels of IL-6 usually reach their peak within a few hours after the insult and remain high for some days [3, 4]. Nevertheless, the shortcoming of this marker is the spontaneous regression even though the critical condition continues; thus, it is not always easy to catch the peak level. Procalcitonin (PCT) is another popular marker of inflammation; plasma levels of this marker begin to increase after those of IL-6, usually within 24 hours after the inflammatory assault, but remain elevated for longer periods of time. CRP is reported to increase shortly after PCT and is sustained longer. While their accuracy and clinical usefulness have been reported [5], none of these conventional biomarkers has, unfortunately, shown satisfactory performance.

Recently, the roles of circulating damage-associated molecular patterns (DAMPs) in the pathophysiology of severe sepsis have attracted much attention [6]. Disorders such as infection and injury amplify the inflammatory response as a part of the self-defense mechanisms, but the overreacted response may concomitantly aggravate the host condition. DAMPs consist of intranuclear or cytosolic substances, such as DNA, histones, and HMGB1, which are released in the course of cell death and act as proinflammatory mediators. For example, high-mobility group protein Box 1 (HMGB1), a chromatin-associated nuclear protein, has been reported to play an important role in the pathophysiology of sepsis, and plasma levels of HMGB1 have been reported as a marker of the severity of sepsis [7]. Some laboratories have reported the usefulness of measuring circulating nuclear proteins as markers of the severity of sepsis [8]. However, we still have quite limited information about the kinetics of circulating DAMPs. Therefore, we carried out this pilot study to elucidate the characteristics of these novel biomarkers and evaluate the usefulness of measuring DAMPs in predicting the outcome of sepsis.

## 2. Materials and Methods

### 2.1. Patient Selection

Patients with severe sepsis admitted to Surugadai Nihon University Hospital were prospectively registered from June 2012 to November 2013. Among these, 30 patients with a known onset time of sepsis were enrolled in this study. The onset was defined as the patients fulfilled the criteria of systemic inflammatory response syndrome (SIRS). Patients who were ≤18 years of age or older than 80 years were excluded. Patients with known chronic kidney disease under hemodialysis were also excluded. The diagnosis of SIRS, sepsis, severe sepsis, and septic shock was made based on the guidelines of the American College of Chest Physicians/Society of Critical Care Medicine Consensus Conference Committee [9]. The study was conducted with the approval of the hospital's Ethics Committee, and the written informed consent was obtained from each of the patients or the next of kin before he/she was included in the study.

### 2.2. Blood Sampling and Measurements

Blood sampling was performed on the day of onset of sepsis (Day 0) and 24 hours (Day 1), 72 hours (Day 3), and 168 hours (Day 7) after the onset of sepsis. Citrated plasma specimens were obtained by centrifugation of the whole blood specimens and stored at −80°C until the assays. The levels of IL-6, PCT, histone H3, nucleosome, and HMGB1 were measured in each of the samples. The Simplified Acute Physiology Score II (SAPS II) [10] was calculated for clinical evaluation of the severity of sepsis. All patients were closely followed up for 28 days.

### 2.3. Quantification of IL-6, PCT, Histone H3, Nucleosome, and HMGB1

IL-6 was measured using a chemiluminescence enzyme immunoassay kit (Fujirebio, Tokyo, Japan). PCT was measured using an electrochemiluminescence immunoassay kit (Roche Diagnostics, Indianapolis, USA).

Histone H3 levels were measured by the originally established sandwich ELISA [11]. Briefly, 20 *μ*L of citrated plasma was added to streptavidin-coated microtiter plates containing biotinylated mouse anti-histone antibody (clone H11-4, Roche Diagnostics) and peroxidase-conjugated anti-histone antibody (rabbit anti-histone H3 antibody [HRP]) (ab53528, Abcam, Cambridge, UK). After the standard washing steps, the peroxidase activity was measured by spectrophotometry.

Nucleosome levels were measured using a sandwich ELISA kit (Cell Death Detection ELISAplus Kit, Roche Diagnostics). HMGB1 levels were measured using an ELISA kit (HMGB1 ELISA Kit II, Shino Test, Tokyo, Japan).

Blood culture analysis was performed with BACTEC 9240 systems (Becton, Dickinson Co., Franklin Lakes, USA) according to the standard procedure.

### 2.4. Receiver Operating Characteristic (ROC) and Other Statistical Analyses

All data in the text and figures are expressed as the means ± standard error (SE). ROC curve analysis was performed to calculate the optimal cutoff levels and to evaluate the areas under the curve (AUC) of each marker to compare their ability to predict the 28-day mortality. Results are reported as the odds ratio (OR), *p* values, and 95% confidence intervals (CI). The above-mentioned analysis was performed using SPSS 22.0 for Windows (IBM SPSS Inc., Chicago, IL). The other statistical analyses were performed using the StatView statistical software package for Windows, version 5.0 (Hulinks, Tokyo, Japan). A repeated-measures analysis of variance (ANOVA) was used for comparison of the continuous variables between two groups. A chi-square test was carried out to compare proportions. The correlations between 2 variables were tested by Pearson's correlation analysis. Statistical differences were deemed significant at *p* < 0.05.

## 3. Results

### 3.1. Patient Demographics

Among the 30 patients, 20 survived (survivor group), while the remaining 10 died (nonsurvivor group). Of the 10 patients of the nonsurvivor group, 3 died within the 7-day study period. There were no significant differences in the age, gender or infection site, positive rate of blood culture, white blood cell count, and blood pressure between the two groups; on the other hand, the baseline SAPS II score was significantly higher in the nonsurvivor group as compared to that in the survivor group (*p* = 0.012) (Table 1).

### 3.2. Time-Course of Changes in the Plasma Levels of the Biomarkers

**Figure 1:**
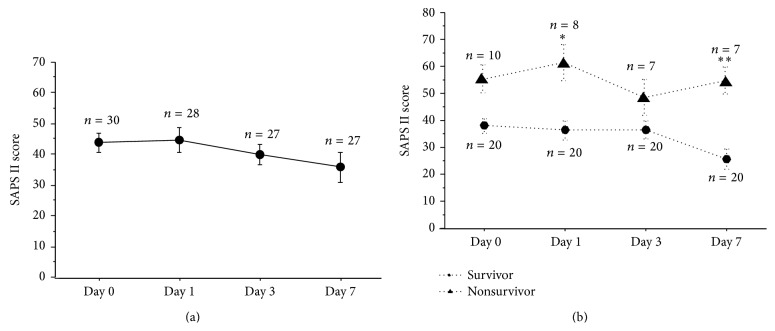
Changes of SAPS II score. SAPS II score was over 40 on Day 0 and the score was sustained over 35 throughout the study period (a). SAPS II score was compared between the survivors group and nonsurvivors group (b). Nonsurvivors group showed significantly higher scores on Day 1 and Day 7. SAPS II: Simplified Acute Physiology Score II, ^*∗*^ *p* < 0.05, ^*∗∗*^ *p* < 0.01.

**Figure 2:**
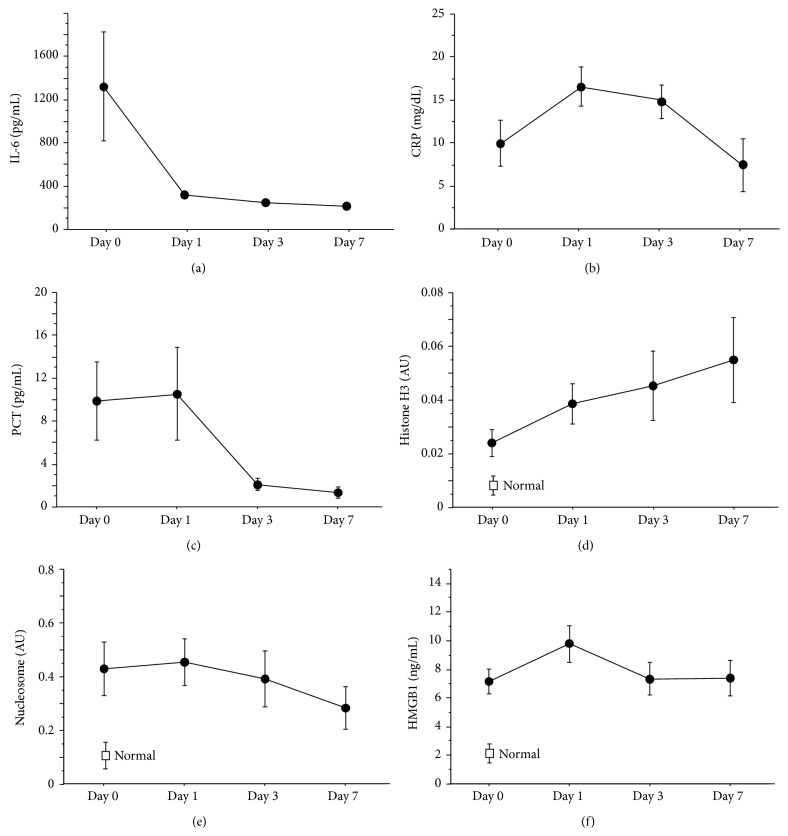
Changes of biomarkers. The time-courses of various biomarkers are plotted in the figure ((a) IL-6, (b) CRP, (c) PCT, (d) histone H3, (e) nucleosome, and (f) HMGB1). The levels of histone H3, nucleosome, and HMGB1 in healthy donors (*n* = 5) were also shown in (d), (e), and (f). The levels of IL-6, CRP, and PCT peaked on Day 1 or Day 3 and decreased thereafter. In contrast, the level of histone H3 increased along with the days and reached its maximum level on Day 7. Data are expressed as means ± standard error. AU: absorbance units, IL-6: interleukin 6, CRP: c-reacting protein, PCT: procalcitonin, and HMGB1: high-mobility group protein Box 1.

The time-course of changes in the mean SAPS II score is plotted in Figure 1(a). The baseline score was higher than 40 and the score remained over 35 throughout the 7-day study period. The plasma IL-6, CRP, and PCT levels reached their peaks on Day 0 or Day 1, and the levels of these markers declined rapidly thereafter (Figure 2). Plasma HMGB1 peaked as early as plasma CRP and PCT; however, the level remained high throughout the 7-day study period. In contrast, the plasma histone H3 level was low on Day 0 and gradually rose to double the baseline level on Day 7. In the case of the plasma level of nucleosome, the level was already elevated on Day 0, with the level gradually declining thereafter during the 7-day study period.

**Figure 3:**
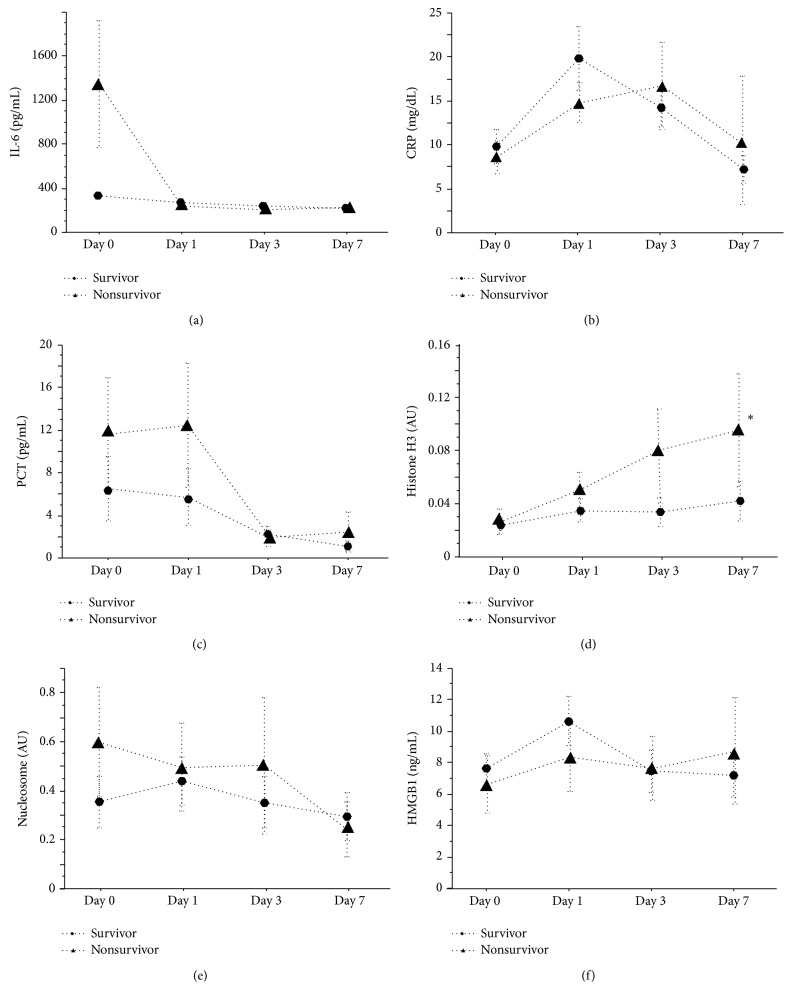
Comparisons of biomarkers between survivors group and nonsurvivors group. The time-courses of various biomarkers in survivors group and nonsurvivors group are plotted ((a) IL-6, (b) CRP, (c) PCT, (d) histone H3, (e) nucleosome, and (f) HMGB1). No difference was observed between the two groups in IL-6, CRP, PCT, nucleosome, and HMGB1, while histone H3 level was higher in nonsurvivors group on Day 7. Data are expressed as means ± standard error. AU: absorbance units, IL-6: interleukin 6, CRP: c-reacting protein, PCT: procalcitonin, and HMGB1: high-mobility group protein Box 1, ^*∗*^ *p* < 0.05.

The time-courses of changes in the SAPS II score (Figure 1(b)) and plasma levels of each of the biomarkers were compared between the survivor group and nonsurvivor group (Figure 3). The SAPS II score was significantly higher in the nonsurvivor group than in the survivor group on Day 1 (*p* < 0.05) and Day 7 (*p* < 0.01). In regard to the plasma levels of IL-6, CRP, PCT, histone H3, nucleosome, and HMGB1, although no statistically significant differences were recognized between the two groups, the plasma IL-6 value on Day 0 was more than 6 times higher in the nonsurvivor group than that in the survivor group. Also, the plasma histone H3 level increased gradually and tended to be higher in the nonsurvivor group on Day 3 (*p* = 0.06) and Day 7 (*p* = 0.04).

**Figure 4:**
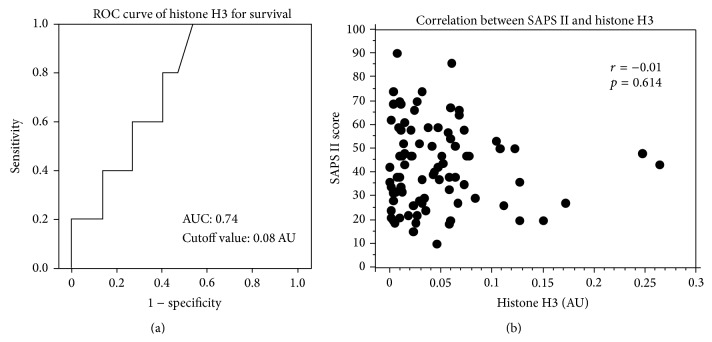
ROC curve for histone H3 for death and correlation between SAPS II and histone H3. ROC curve analysis calculated AUC for histone H3 on Day 7 for death. AUC was 0.740 (95% CI: 0.513–0.967). The cutoff value of the histone H3 was 0.08 AU (a). No significant correlation between SAPS II score and histone H3 level on Day 7 was recognized (*r* = −0.01, *p* = 0.614 (b)). SAPS II: Simplified Acute Physiology Score II, ROC: receiver operating characteristic, AUC: area under the curve, OR: odds ratio, CI: confidence intervals, and AU: absorbance units.

AUCs for IL-6, CRP, PCT, histone H3, nucleosome, and HMGB1 on Day 7 for survival were 0.693 (95% CI: 0.435–0.952), 0.501 (95% CI: 0.287–0.882), 0.667 (95% CI: 0.375–0.958), 0.740 (95% CI: 0.513–0.967, Figure 4(a)), 0.571 (95% CI: 0.296–0.847), and 0.580 (95% CI: 0.225–0.935), respectively. The cutoff value of the histone H3 calculated by the ROC curve analysis was 0.08 AU. No significant correlation between SAPS II score and histone H3 level was recognized (*r* = −0.01, *p* = 0.614).

**Figure 5:**
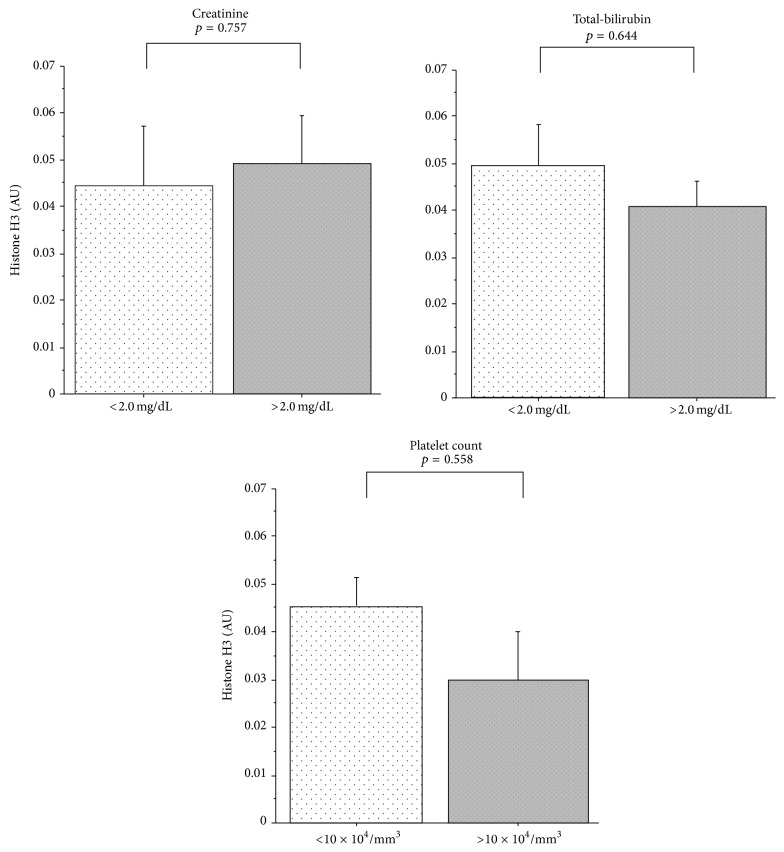
Comparison of histone H3 levels in patients with or without organ dysfunction. Circulating levels of histone H3 in patients with renal dysfunction (creatinine > 2.0 mg/dL), hepatic dysfunction (total-bilirubin > 2.0 mg/dL), and thrombocytopenia (platelet count < 10 × 10^4^/mm^3^) are compared to those in the patients without organ dysfunction. Significant difference was not seen between patients with and without organ dysfunction. Data are expressed as mean ± standard error. AU: absorbance units.

Figure 5 demonstrates the relationship between organ dysfunction and the plasma histone H3 level. The histone H3 levels did not differ between the groups with and without liver dysfunction, renal dysfunction, or thrombocytopenia.

## 4. Discussion

Severe sepsis is the leading nosocomial cause of death, with a mortality rate as high as 50% [12]. Although the management of severe sepsis has improved remarkably over the years, the lack of reliable markers of the severity limits further advance. It is not always difficult to decide the induction of the bundle care recommended by the Surviving Sepsis Campaign [13], as most cases exhibit significant increases in the plasma levels of the inflammatory biomarkers such as IL-6 and PCT. Nevertheless, it is also true that these conventional biomarkers do not elevate in certain cases, and such phenomena are often seen when we miss the initial elevation. To avoid missing the elevation of biomarkers, we selected patients in whom the date of onset of sepsis was accurately determined in this study, and we found that the plasma IL-6 and PCT reached their peak in the early stage of sepsis as reported before [14, 15] and declined rapidly by Day 3. In case of CRP, it reached the peak level on Day 1 and was sustained longer. The reported cutoff values of IL-6 and PCT for the severity are 500 pg/mL [3] and 6.6 ng/mL [16], respectively, and none of the patients in either the survivor group or the nonsurvivor group showed IL-6 and PCT values higher than the cutoff levels on Day 3. These results indicate that the transient elevation of IL-6 and PCT reflects the temporal reaction in the early stage of sepsis, and their plasma levels decrease after the initial response irrespective of the severity of sepsis. Therefore, it will be more clinically useful to identify other parameters whose plasma levels would remain sustained for longer periods of time.

DAMPs are passively released from damaged or dead cells and propagate further inflammatory reaction in the host. In this study, we measured the sequential changes in the plasma levels of histone H3, nucleosome, and HMGB1 as DAMPs. It is reported that histone and nucleosome are extruded from damaged cells [17]. In contrast, HMGB1 is not only released from the damaged cells but also secreted actively [6]. The results of this study showed that the circulating levels of DAMPs remain higher for much longer and move more in parallel with the SAPS II scores. Interestingly, the plasma level of histone H3 increased over time, especially in the nonsurvivor group and the AUC for the death was the highest among the markers examined in this study. However, we could not find significant relationship between histone H3 and SAPS II score. This is probably because there is time lag between the changes of SAPS II score and the elevations of histone H3. Histones released from the necrotic cells induce organ dysfunction by exerting tissue toxicity [18] and inducing thrombus formation through the activation of platelets [19]. The clinical significance of circulating histones has only been reported by a few studies [20, 21]. Ekaney et al.'s report [21] is the only report so far that suggests the existence of a correlation between the plasma histone level and the severity of sepsis. One of the reasons may be the absence of a reliable assay for plasma histone. Some laboratories are intensively engaged in trying to establish a valid assay; however, none is in practical use at present.

Nucleosomes are complexes formed by DNA and histones. They are mainly released from apoptotic cells under normal physiologic conditions and are eliminated mostly by phagocytes [8]. High rates of cell death are associated with higher concentrations of nucleosomes in the circulation [22] and several groups reported that measurement of the circulating levels of nucleosomes is useful for predicting the prognosis of sepsis [7, 23]. In the current study, the plasma nucleosome levels remained elevated until Day 3 but declined thereafter, and the difference in the levels between the two groups was lower than that in the levels of histone H3. Since we expected these two DAMPs to change similarly, further study is required for the validation.

As for the metabolism of circulating DAMPs, the mechanism has not been fully clarified. In this study, the levels were not affected by organ dysfunction of the liver, kidney, and coagulation.

Finally, there were some limitations of this study. This was a pilot study and the number of patients was quite limited. Notwithstanding, this study suggests that the measurement of plasma histone is helpful for monitoring the severity of sepsis. This finding needs to be confirmed in a larger number of patients.

## 5. Conclusion

We examined the changes in the plasma levels of various biomarkers in 30 patients with sepsis and also the time-course of changes in the plasma levels of each of the markers. In contrast to the rapid decrease of the plasma IL-6 and PCT levels, elevated levels of DAMPs such as histone H3, nucleosome, and HMGB1 were sustained for longer period of time. Furthermore, the elevated plasma levels of histone H3 seemed to be associated with the severity of sepsis.

## Figures and Tables

**Table 1:** Patient demographics.

	Survivor group (*n* = 20)	Nonsurvivor group (*n* = 10)	*p* value
Age (y.o.)	56 ± 3 [27–78]	64 ± 5 [35–79]	0.147
Gender: male/female	12/8	7/3	0.866
Infection site			
Abdominal	2	0	
Lung	7	7	
Urinary	3	1	
Blood	4	1	
Unknown	4	1	
Treatment			
Operation	1	3	
Ventilation	8	5	
Hemofiltoration	3	2	
Blood culture positive (%)	3 (15)	4 (40)	0.312
Gram-positive coccus	5	2	
Gram-negative bacillus	0	2	
Others	1	1	
Baseline SAPS II score	38 ± 3 [21–59]	59 ± 6 [29–86]	0.012
